# Increased growth ability and pathogenicity of American- and Pacific-subtype Zika virus (ZIKV) strains compared with a Southeast Asian-subtype ZIKV strain

**DOI:** 10.1371/journal.pntd.0007387

**Published:** 2019-06-06

**Authors:** Yasuhiro Kawai, Eri Nakayama, Kenta Takahashi, Satoshi Taniguchi, Ken-ichi Shibasaki, Fumihiro Kato, Takahiro Maeki, Tadaki Suzuki, Shigeru Tajima, Masayuki Saijo, Chang-Kweng Lim

**Affiliations:** 1 Division of Biosafety Control and Research, National Institute of Infectious Diseases, Shinjuku, Tokyo, Japan; 2 Department of Virology I, National Institute of Infectious Diseases, Shinjuku, Tokyo, Japan; 3 Inflammation Biology Group, QIMR Berghofer Medical Research Institute, Brisbane, Queensland, Australia; 4 Department of Pathology, National Institute of Infectious Diseases, Shinjuku, Tokyo, Japan; Institute for Disease Modeling, UNITED STATES

## Abstract

We investigated the growth properties and virulence in mice of three Zika virus (ZIKV) strains of Asian/American lineage, PRVABC59, ZIKV/Hu/Chiba/S36/2016 (ChibaS36), and ZIKV/Hu/NIID123/2016 (NIID123), belonging to the three distinct subtypes of this lineage. The American-subtype strain, PRVABC59, showed the highest growth potential in vitro, whereas the Southeast Asian-subtype strain, NIID123, showed the lowest proliferative capacity. Moreover, PRVABC59- and NIID123-infected mice showed the highest and lowest viremia levels and infectious virus levels in the testis, respectively, and the rate of damaged testis in PRVABC59-infected mice was higher than in mice infected with the other two strains. Lastly, ZIKV NS1 antigen was detected in the damaged testes of mice infected with PRVABC59 and the Pacific-subtype strain, ChibaS36, at 2 weeks post-inoculation and in the epididymides of PRVABC59-infected mice at 6 weeks post-inoculation. Our results indicate that PRVABC59 and ChibaS36 exhibit increased abilities to grow in vitro and in vivo and to induce testis damage in mice.

## Author summary

Zika virus (ZIKV) is classified into two lineages, African and Asian/American. Phylogenetic analyses have revealed that Asian/American-lineage ZIKV strains can be divided into three distinct subtypes, the American, Pacific, and Southeast Asian subtypes, presenting several amino acid differences. In this study, we examined the in vitro and in vivo growth of three Asian/American lineage ZIKV strains belonging to the three subtypes. The American-subtype strain and the Southeast Asian-subtype strain exhibited the highest and lowest growth potential in vitro, respectively, and mice infected with these ZIKV strains also showed the highest and lowest viremia levels and infectious virus levels in the testis. Moreover, the rate and extent of testis damage were highest in mice infected with the American-subtype strain. Our results indicate that the American-subtype and Pacific-subtype strains exhibit increased ability to grow in vitro and in vivo and to induce testis damage in mice.

## Introduction

Zika virus (ZIKV; genus: *Flavivirus*; family: *Flaviviridae*) was first isolated from a sentinel rhesus monkey in the Zika forest of Uganda in 1947 [1]. ZIKV infection in humans was first identified in Uganda and the United Republic of Tanzania in 1952, and human ZIKV infections (ZIKV disease, ZVD) have been sporadically detected in Africa and Asia for > 50 years since the initial isolation [2]. The first large outbreak of ZIKV infection in humans was identified in Yap Island in the Federal State of Micronesia in 2007 and a ZIKV-infection outbreak was also confirmed in French Polynesia in the South Pacific in 2013, with an estimated number of 30,000 ZVD cases [3–5]. In 2014–2015, ZIKV-infection epidemics spread to other Pacific regions and to the Americas. In 2016, patients with ZVD were reported in several countries in Southeast Asia, including Singapore, Thailand, Vietnam, and the Philippines. ZIKV is transmitted to humans mainly through the bite of *Aedes* mosquitoes and non-vector transmission of ZIKV has also been reported to occur through blood transfusion, transplantation, and sexual intercourse [6–8].

The clinical symptoms caused by ZIKV infection are generally self-limiting and include fever, rash, headache, joint and muscle pain, and conjunctivitis. Approximately 60–70% and 90% of symptomatic ZIKV-infected patients develop fever and rash, respectively. When pregnant women are infected with ZIKV, the fetus can be infected with ZIKV through the placenta, which can cause congenital neurological malformations with the following symptoms: microcephaly, sensorineural abnormalities, cerebral calcification, and abortion [9, 10]. ZIKV infection also causes a severe neurological complication, Guillain–Barré syndrome, and ZIKV has demonstrated the ability to infect diverse cell types including neuronal cells [9, 11, 12].

The ZIKV genome is comprised of a single-stranded, positive-sense RNA that encodes three structural proteins (C, prM, and E) and seven non-structural proteins (NS1, NS2A, NS2B, NS3, NS4A, NS4B, and NS5) in one open reading frame [13]. ZIKV is classified into two lineages, African and Asian/American; the recent epidemics associated with severe neurological and congenital abnormalities in Pacific regions and the Americas were caused by the spread of Asian/American-lineage ZIKV from Southeast Asia [14, 15]. Phylogenetic analyses performed using complete ZIKV genomes indicate that ZIKV strains in the Asian/American lineage can be divided into three subtypes, American, Pacific, and Southeast Asian, which present several differences in their amino acid sequences [16, 17]. Studies conducted using a mouse model showed that American-subtype ZIKV strains induce more severe neurological disorders and marked immune responses in mice compared with Southeast Asian-subtype strains [18, 19]. These findings raise the possibility that recent genetic changes in the ZIKV genome altered the properties of the virus, such as virulence and tissue tropism, and also contributed to the spread of the ZIKV endemic area and the increase in the cases of congenital ZIKV infections in Pacific regions and the Americas [17]. Accordingly, mutations in prM and NS1 were reported to be involved in the differences between Asian/American-lineage strains in terms of pathogenicity in mice, viral protein antigenemia, and interferon induction in host cells [18, 20–24]. However, only a limited number of Asia/American-lineage ZIKV strains were previously used for evaluating virulence; thus, further analyses must be conducted using additional ZIKV isolates to elucidate the relationship between genetic variation and pathogenicity among the Asian/American-lineage strains [25].

In this study, we examined the in vitro and in vivo growth of three ZIKV strains that belong to the three distinct subtypes in the Asian/American lineage. Recent studies have demonstrated that Asian/American lineage ZIKV infection in mice is not lethal, but causes testis damage; this indicates that testis damage in ZIKV-infected mice can be used as an index for assessing ZIKV pathogenicity [26–33]. Therefore, in this study we pathologically evaluated the male reproductive organs of mice infected with each of the three strains to determine the differences in pathogenicity among these ZIKV strains.

## Methods

### Ethics statement

Mouse experiments were performed in biosafety level 2 animal facilities, in accordance with the “Guidelines for Animal Experiments Performed at the National Institute of Infectious Diseases (NIID),” under approval (no. 116067) from the Animal Welfare and Animal Care Committee of the NIID, Japan. All efforts were made to minimize any potential pain and distress. Mice infected with ZIKV were observed daily for adverse reactions and signs of diseases. For collection of organ samples, mice were euthanized by using isoflurane.

The ZIKV strains used in this study were previously published and we did not obtain samples from patients specifically for this study [16, 34, 35].

### Viruses, cell culture, plaque assay, and growth kinetics assay

We used four ZIKV strains: MR766-NIID (MR766; accession no. LC002520), ZIKV/Hu/Chiba/S36/2016 (ChibaS36; accession no. LC191864) [35], PRVABC59 (accession no. KU501215), and ZIKV/Hu/NIID123/2016 (NIID123; accession no. LC219720) [16] (Table 1).

**Table 1:** Zika virus strains used in this study.

Strain name	Lineage	Year isolated	Country (region)	Source	Accession no.	Passage history (no. of passages)
MR766-NIID	African	1947	Uganda	Rhesus monkey	LC002520	Mouse brain (150?), C6/36 cells (1), Vero cells (2)
PRVABC59	Asian /American	2015	USA (Puerto Rico)	Human	KU501215	Vero cells (4)
ZIKV/Hu /Chiba/S36 /2016	Asian /American	2016	Fiji	Human	LC191864	Vero cells (4)
ZIKV/Hu /NIID123 /2016	Asian /American	2016	Vietnam	Human	LC219720	C6/36 cells (1), Vero cells (7)

ChibaS36 and NIID123 were originally isolated from, respectively, a patient infected with ZIKV in Fiji in 2016 and a patient infected with ZIKV in Vietnam in 2016 [16, 34, 35]. PRVABC59 was kindly provided by Dr. Beth Bell of the US CDC. MR766 was maintained at the NIID, Japan; this strain was used as a positive control when evaluating the growth ability of Asian/American-lineage ZIKV in mice [34]. These viruses were propagated in Vero cells (strain 9013). Vero cells and mosquito-derived C6/36 cells were cultured at 37 °C and 28 °C, respectively, under 5% CO_2_ in Eagle’s minimum essential medium (MEM) supplemented with 10% heat-inactivated fetal bovine serum (FBS) and 100 U/mL penicillin–streptomycin (MEM-10FBS). The infectious titer of the viruses was determined using the plaque assay, as previously described [36]. Growth kinetics was analyzed in Vero cells and C6/36 cells as previously described [34]. Briefly, Vero and C6/36 cells were plated in 6-well culture plates (3 × 10^5^ cells/well) and infected with ZIKV at a multiplicity of infection of 0.01 plaque-forming units (PFU) per cell. Small aliquots of the culture medium were collected periodically and the titer of the infectious ZIKV in each aliquot was determined using a plaque assay performed on Vero cells. Growth curves were statistically compared using BellCurve for Excel (Social Survey Research Information, Tokyo, Japan) employing a two-way ANOVA test.

### Phylogenetic analysis and comparison of amino acid sequences

To construct a phylogenetic tree, the nucleotide sequences of 33 ZIKV strains were aligned and analyzed using the maximum likelihood method with 1,000 bootstrap replicates using the MEGA7 program [37]. Complete amino acid sequences of the ZIKV strains were aligned using GENETYX gene-analysis software (Genetyx Corp., Tokyo, Japan).

### Mouse challenge and sample collection

Interferon-α/β receptor-1 gene-knockout (IFNAR1-KO) C57BL/6 mice were produced, bred, and maintained in a specific-pathogen-free environment as previously described [34, 38, 39]. Mice (8–16 weeks old) were inoculated with 1 × 10^4^ PFU of each virus in culture medium diluted with MEM supplemented with 2% FBS (MEM-2FBS) through the subcutaneous route in the footpad. Tissue and fluid samples (serum, brain, spinal cord, kidney, spleen, testis, epididymis, sperm, and epididymal fluid) were collected from the ZIKV-infected mice and the infectious virus and viral RNA levels in the samples were measured. Tissue weights were also determined prior to homogenate preparation. To prepare sperm and epididymal fluid samples, cauda epididymis was placed in a microtube containing 100 μL of phosphate-buffered saline (PBS) and incised 4–5 times with scissors to allow the sperm to swim out and disperse, after which the cauda epididymis was removed and the liquid phase was centrifuged for 5 min at 10,000 × g. The supernatant was recovered as the epididymal fluid and the precipitate was resuspended in 100 μL of PBS and used as the sperm sample. The collected tissues (10–200 mg) were homogenized in 500 μL of MEM-2FBS and then used for further analyses.

### Measurement of viral titer

We determined the 50% tissue-culture infective dose (TCID_50_) for each organ sample as previously described [30, 40]. Briefly, to pre-amplify infectious ZIKV in the organ samples using C6/36 cells, the organ samples were serially diluted (1:10–1:10^8^) with MEM-2FBS and the C6/36 cells cultured in 96-well plates were then inoculated with each sample and incubated for 5 days at 28 °C. The pre-amplification step was added to improve the detection of low-level infectious viruses. Subsequently, 25-μL aliquots of the culture supernatants were transferred to Vero cells cultured in 96-well plates and incubated for 5 days at 37 °C. Lastly, the cells were fixed in 10% formaldehyde solution and stained with Methylene Blue solution to visualize the cytopathic effect induced by the ZIKV infection. Viral titers were statistically compared using either BellCurve for Excel employing the Mann-Whitney U test or SPSS (IBM, Chicago, IL, USA) employing the repeated-measures ANOVA test.

### Measurement of viral-genome copy number using real-time RT-PCR

Total RNA was extracted from each organ sample using a High Pure Viral RNA Purification Kit (Roche Diagnostics, Indianapolis, IN, USA). ZIKV RNA was quantified by performing quantitative real-time RT-PCR with Fast Virus One-Step Master Mix (Thermo Fisher Scientific, Waltham, MA, USA). The ZIKV genome was amplified using the following primers: ZIKV 1086 (5′-CCGCTGCCCAACACAAG-3′), ZIKV 1162c (5′-CCACTAACGTTCTTTTGCAGACAT-3′), and Probe ZIKV 1107-FAM (5′FAM-AGCCTACCTTGACAAGCAGTCAGACACTCAA-TAMRA3′) [14]. Genome copy numbers were statistically compared using BellCurve for Excel employing the Mann-Whitney U test.

### Histological and immunohistochemical analyses

Testes and epididymides were fixed in 10% phosphate-buffered formalin, embedded in paraffin, sectioned, and stained with hematoxylin and eosin. Immunohistochemistry was performed using an anti-ZIKV NS1 antibody (C01886G, Meridian Bioscience, Cincinnati, OH, USA) as the primary antibody. Specific antigen–antibody reactions were visualized by 3,3-diaminobenzidine tetrahydrochloride staining using a DAKO LSAB2 system (DAKO Cytomation, Glostrup, Denmark).

## Results

### Phylogenetic analysis of three Asian/American-lineage ZIKV strains: PRVABC59, ZIKV/Hu/Chiba/S36/2016, and ZIKV/Hu/NIID123/2016

**Fig 1:**
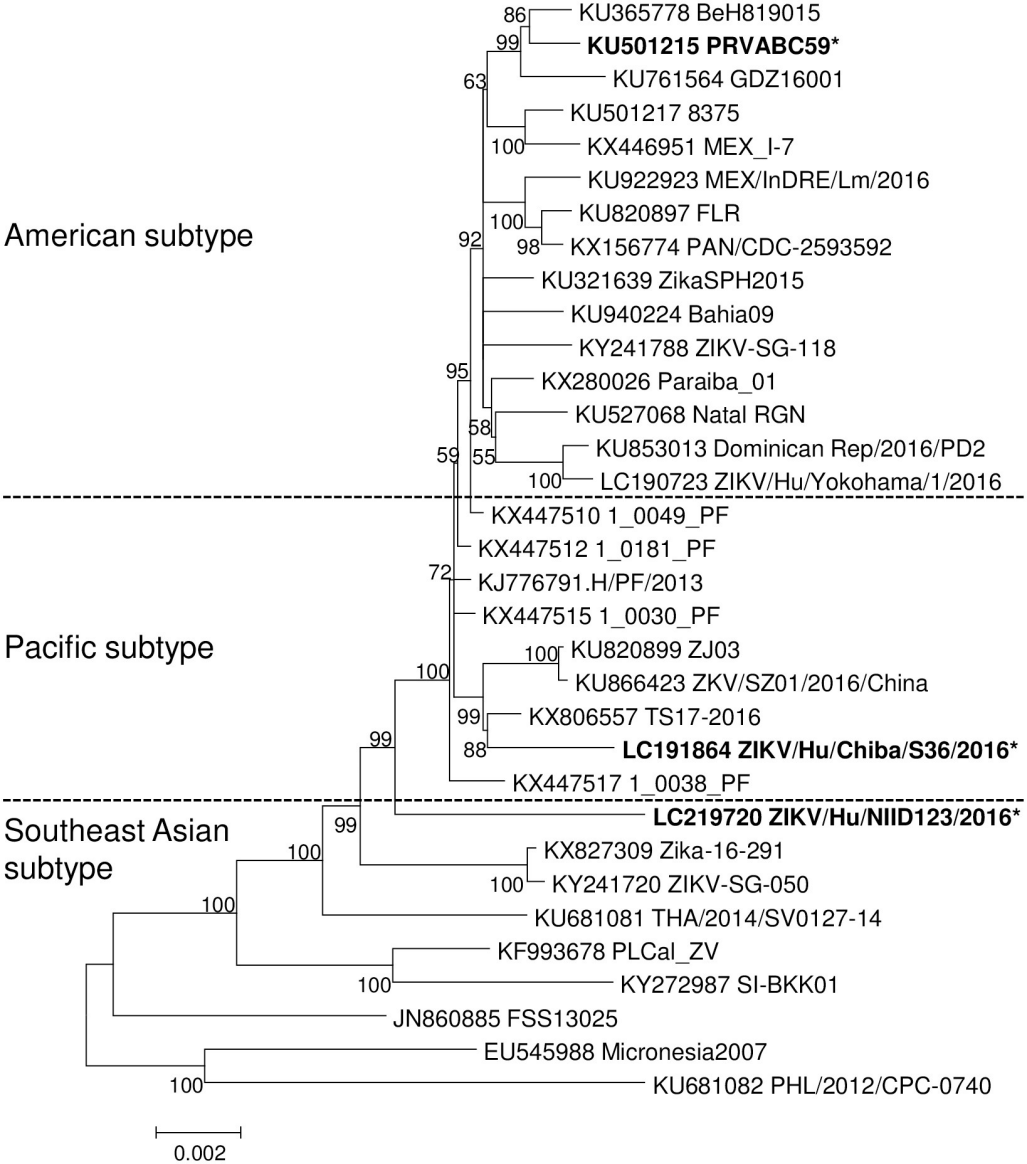
Phylogenetic analysis of Asian/American-lineage ZIKV strains based on the nucleotide sequences of the entire viral genome. Numbers at branch points: bootstrap values (1,000 replicates); asterisks: strains used for characterization in this study. The strains used for construction of the tree are listed in S1 Table.

The phylogenetic analysis results indicated that the three ZIKV strains, PRVABC59, ChibaS36, and NIID123, belong to distinct subtypes in the Asian/American lineage: PRVABC59, American subtype; ChibaS36, Pacific subtype; and NIID123, Southeast Asian subtype (Fig 1).

### In vitro growth of African- and Asian/American-lineage ZIKV strains

**Fig 2:**
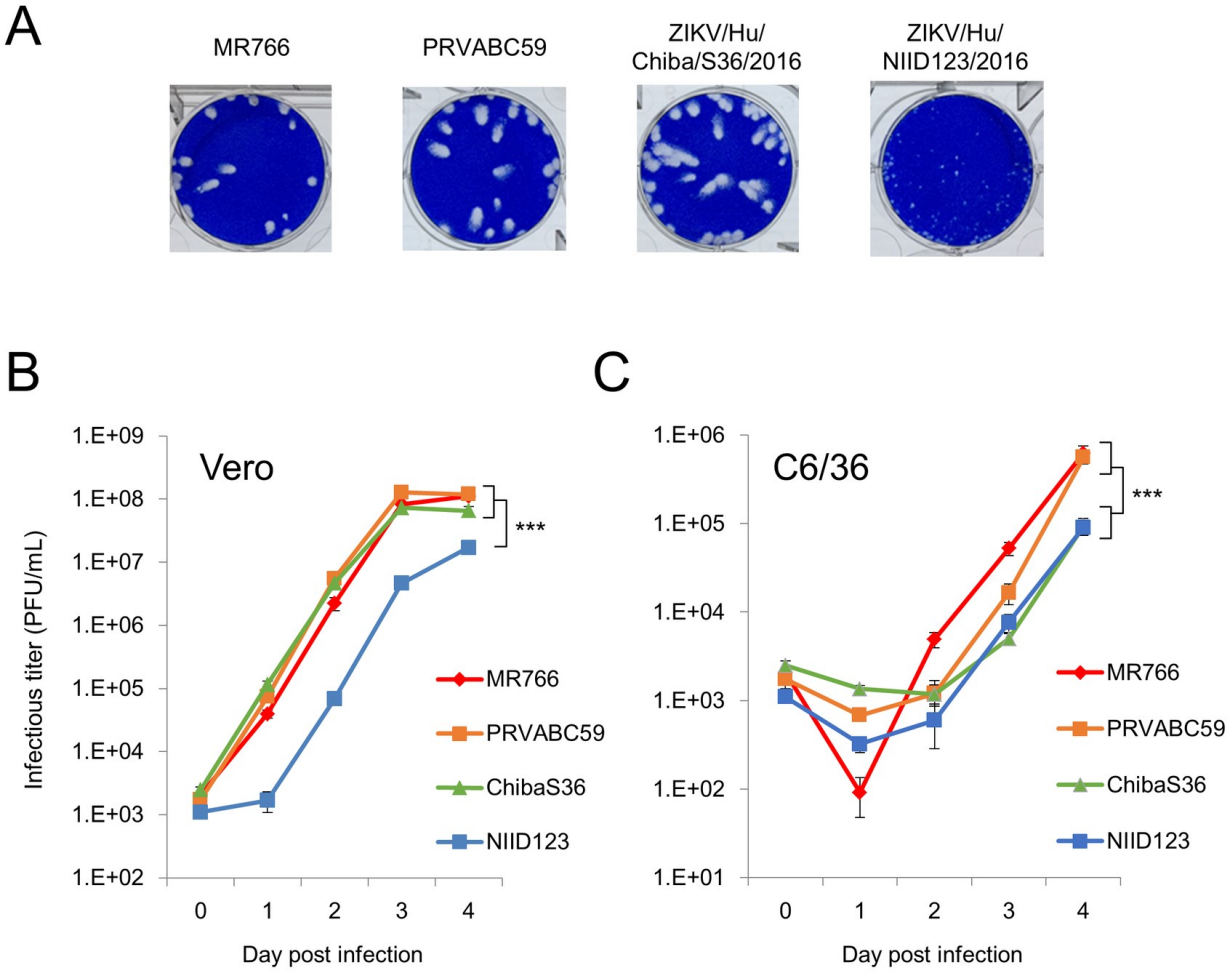
In vitro growth of four ZIKV strains: MR766-NIID, PRVABC59, ZIKV/Hu/Chiba/S36/2016, and ZIKV/Hu/NIID123/2016. A) Plaques formed in Vero cells following infection with each strain. B and C) Growth curves of the strains following infection of Vero cells (B) and mosquito C6/36 cells (C). Cells were added to 6-well culture plates and infected with the strains at a multiplicity of infection of 0.01 PFU/cell. Values represent means ± standard deviation from three independent experiments. Significance was analyzed using two-way ANOVA (***P < 0.001).

The plaques formed on Vero cells by NIID123 were clearly smaller than those formed by each of the other three strains (Fig 2A). NIID123 also replicated more slowly than the other three ZIKV strains in Vero cells (Fig 2B). The growth kinetics of NIID123 and ChibaS36 in C6/36 cells revealed a slower replication rate than that of MR766 and PRVABC59 (Fig 2C).

### Growth of African- and Asian/American-lineage ZIKV strains in IFNAR1-KO mice

**Fig 3:**
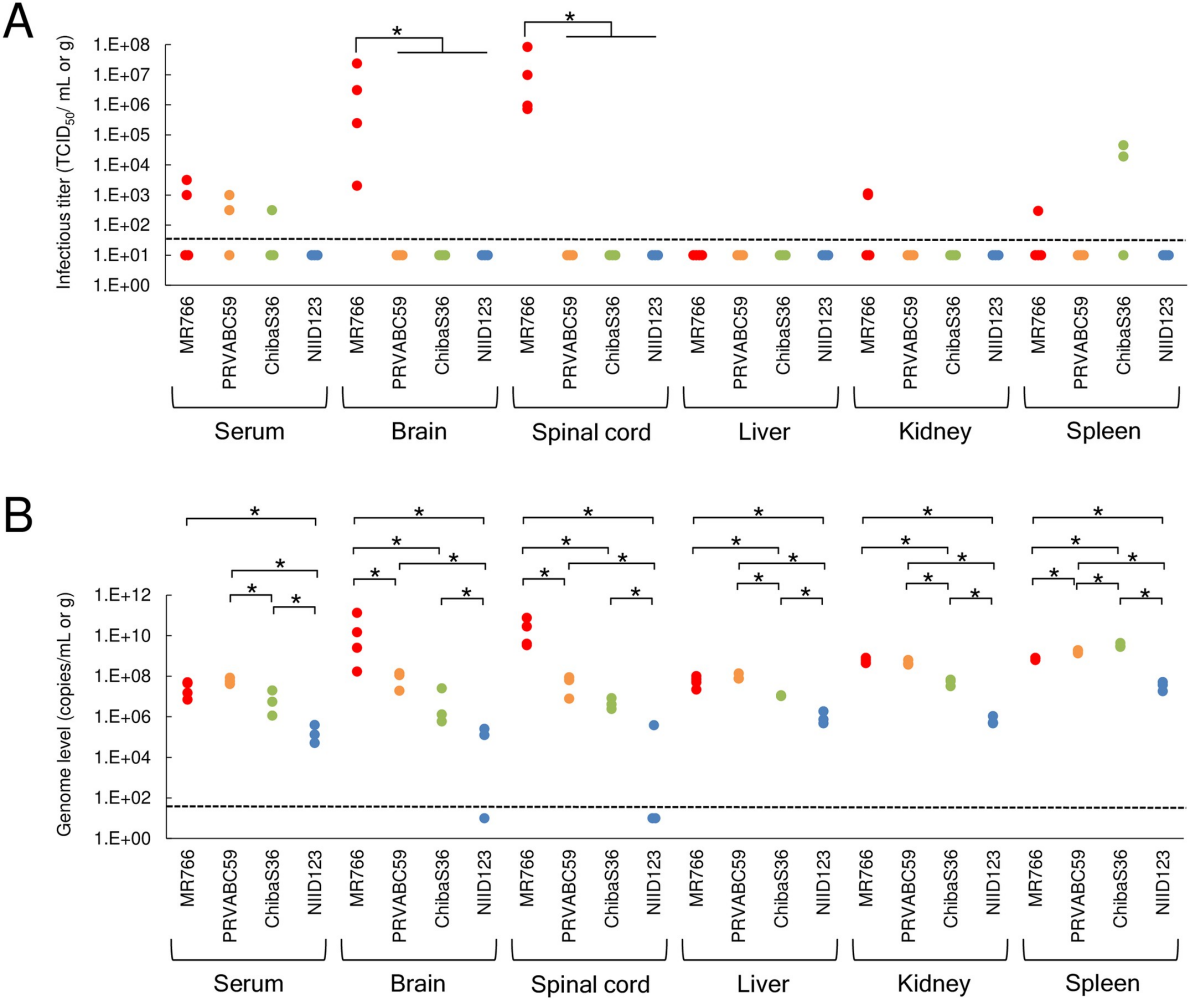
Levels of viremia/infectious virus and viral RNA in ZIKV-infected IFNAR1-KO mice. Mice infected with MR766-NIID (n = 4), PRVABC59 (n = 3), ZIKV/Hu/Chiba/S36/2016 (n = 3), and ZIKV/Hu/NIID123/2016 (n = 3) were euthanized at 6 days post-inoculation and the blood, brain, spinal cord, liver, kidney, and spleen were collected. Sera and organ homogenates were prepared from the specimens and then used for determining the infectious virus titer (TCID_50_/mL or g) and quantifying the viral genome (genome copies/mL or g). A) Infectious virus titers in sera and tissues and B) copy number of viral genome in sera and tissues. Dotted line: detection limit. Significance was analyzed using the Mann-Whitney U test (*P < 0.05).

No statistically significant differences in viremia levels were observed among the four ZIKV-infected mouse groups at 6 days post-inoculation (Fig 3A). However, no infectious virus was detected in the serum of any mice infected with NIID123. Viral RNA was detected in all mice infected with each strain; however, the viral RNA level in the NIID123-infected mice was the lowest among the four groups (Fig 3B). Infectious virus was detected in the brain and spinal cord of all mice infected with MR766, but not in any of the mice infected with the Asian/American-lineage strains (Fig 3A). No infectious virus was detected in the liver and kidney of most of the mice infected with each of the four ZIKV strains. Infectious virus was detected in the spleen in 2/3 of ChibaS36-infected mice, but not in mice infected with PRVABC59 or NIID123. Viral RNA was detected in all tissues of mice infected with MR766, PRVABC59, or ChibaS36 (Fig 3B). Viral RNA was not detected in the brain or spinal cord of 50% of the mice infected with NIID123. Furthermore, the viral RNA level in the tissues of mice infected with NIID123 was the lowest among the four groups.

**Fig 4:**
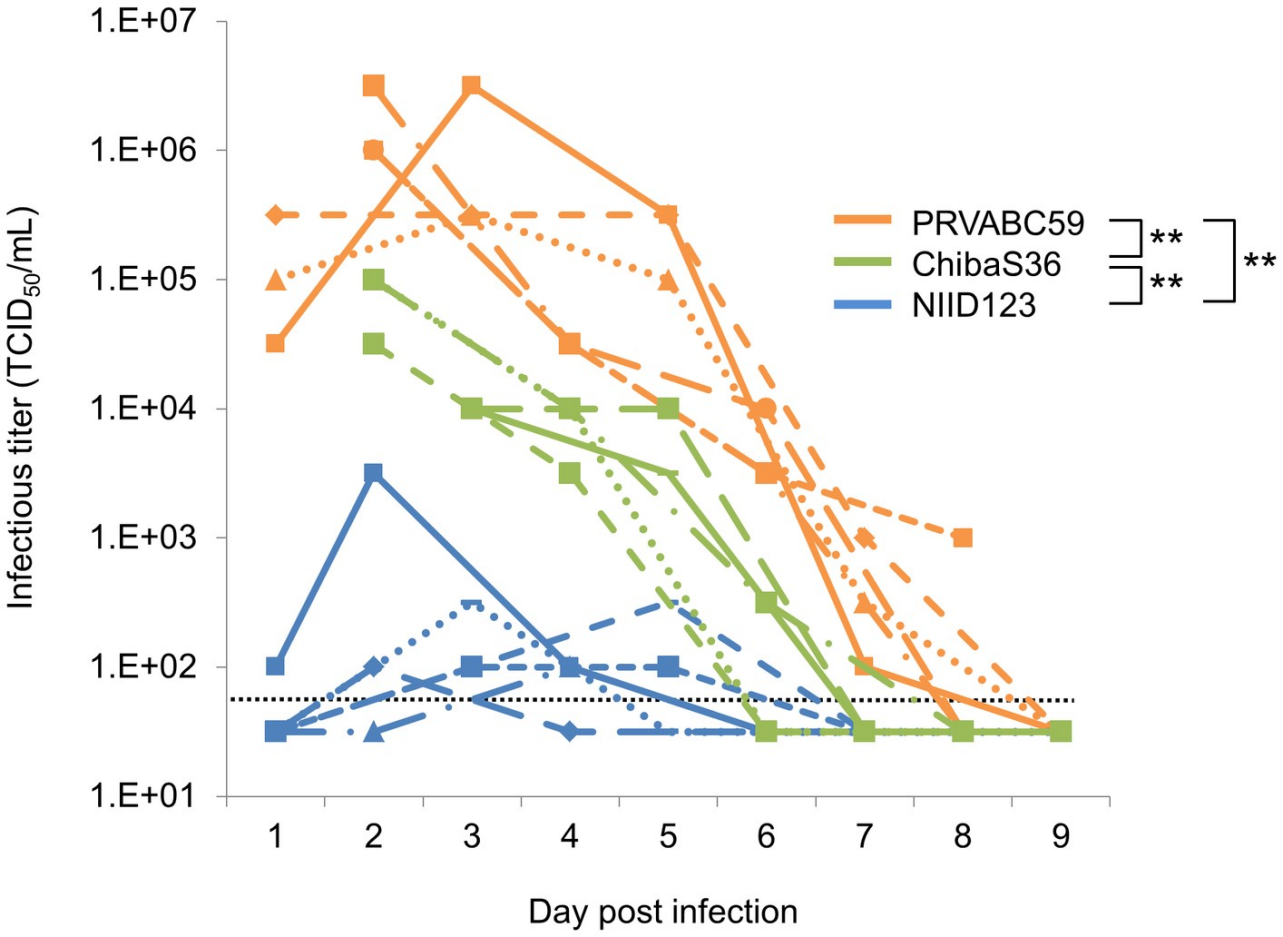
Viremia levels in ZIKV-infected IFNAR1-KO mice. Mice inoculated with the three Asian/American strains, PRVABC59 (n = 6), ZIKV/Hu/Chiba/S36/2016 (n = 6), and ZIKV/Hu/NIID123/2016 (n = 6), were divided into two groups (three mice/group) and blood was collected at the indicated time points at 2-day intervals: (1,) 3, 5, 7, and 9 days post-infection for group 1; and (1,) 2, 4, 6, and 8 days post-infection for group 2. Sera were prepared and used for quantifying the infectious virus titer (TCID_50_/mL). Dotted line: detection limit. Significance was analyzed by performing repeated-measures ANOVA with the TCID_50_ data from 2 to 8 days post-infection (**P < 0.01).

Next, we analyzed the three Asian/American-lineage ZIKV in further detail. The time course of change in viremia levels in mice infected with the three strains was also examined (Fig 4). Mice infected with ChibaS36 showed significantly lower and higher viremia levels compared with mice infected with PRVABC59 and NIID123, respectively. All inoculated mice survived to the final collection point (8 or 9 days post-infection).

### Growth of Asian/American-lineage ZIKV strains in the male genital tract of IFNAR1-KO mice

**Fig 5:**
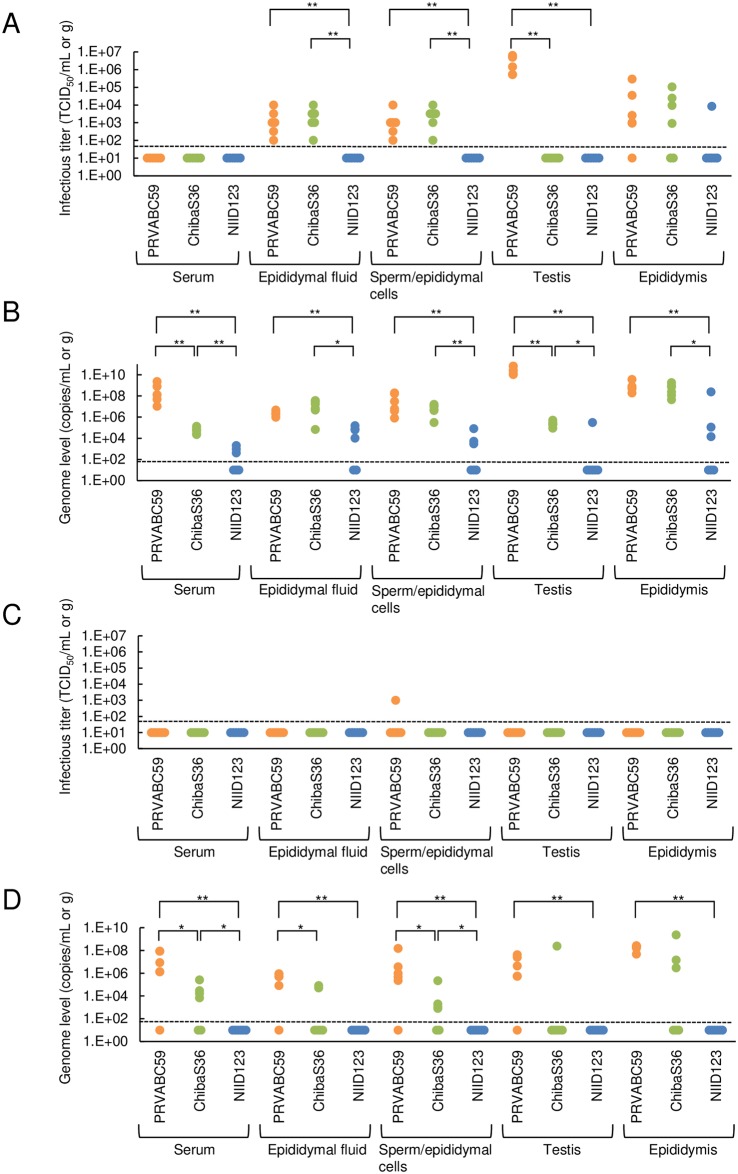
Viremia/infectious virus and viral RNA levels in serum, epididymal fluid, epididymal cells including sperm (sperm/epididymal cells), testis, and epididymis of ZIKV-infected IFNAR1-KO mice. Mice inoculated with the Asian/American-lineage strains PRVABC59 (n = 6), ZIKV/Hu/Chiba/S36/2016 (n = 6), and ZIKV/Hu/NIID123/2016 (n = 6) were euthanized at 2 weeks (A and B) or 6 weeks (C and D) post-inoculation and serum, epididymal fluid, sperm/epididymal cell, testis, and epididymis samples were collected. Sera, epididymal fluid, sperm/epididymal cell, and tissue homogenates were used to quantify the infectious virus titer (TCID_50_/mL or g) and the viral genome (genome copies/mL or g). (A and C) infectious virus titers and (B and D) copy numbers of the viral genome in serum, epididymal fluid, sperm/epididymal cells, testis, and epididymis of mice at 2 weeks (A and B) and 6 weeks (C and D) post-inoculation. Dotted line: detection limit. Significance was analyzed using the Mann-Whitney U test (*P < 0.05, **P < 0.01).

All inoculated mice had survived at 2 weeks post-inoculation. No infectious virus was detected in the serum in any of the mice infected with each Asian/American-lineage ZIKV strain (Fig 5A). Infectious virus was detected at similar levels in the epididymal fluid and epididymal cells including sperm (sperm/epididymal cells) samples from all mice infected with PRVABC59 or ChibaS36, whereas infectious ZIKV was not detected in the samples from NIID123-infected mice. Infectious virus was detected in the testes of all PRVABC59-infected mice, but not in the testes of ChibaS36- or NIID123-infected mice. Lastly, infectious virus was detected in the epididymis of some of the mice infected with each strain, but no significant difference was observed between the three groups.

Viral RNA was detected in all specimens collected from PRVABC59- and ChibaS36-infected mice at 2 weeks post-inoculation (Fig 5B). Serum viral RNA levels were significantly higher in PRVABC59-infected mice than in ChibaS36-infected mice. Viral RNA was also detected in the serum of NIID123-infected mice, but the levels were significantly lower than those in the serum of PRVABC59- and ChibaS36-infected mice. Viral RNA levels in the testis were significantly higher in PRVABC59-infected mice than in ChibaS36-infected mice, whereas viral RNA levels in the epididymis, epididymal fluid, and sperm/epididymal cell samples from PRVABC59-infected mice were similar to those in the corresponding samples from ChibaS36-infected mice.

All inoculated mice survived at 6 weeks post-inoculation. Infectious virus was not detected in any of the serum samples and most of the genital samples collected from mice infected, except in the sperm/epididymal cell specimen from one PRVABC59-infected mouse (Fig 5C). Viral RNA was identified in the serum, epididymal fluid, sperm/epididymal cell, testis, and epididymis specimens of PRVABC59-infected mice and ChibaS36-infected mice (Fig 5D). The levels of viral RNA in the serum, epididymal fluid, and sperm/epididymal cell specimens of PRVABC59-infected mice were higher than the levels in the specimens of ChibaS36-infected mice. No viral RNA was detected in any of the specimens of NIID123-infected mice.

### Histological and immunohistochemical analyses of testis and epididymis in mice infected with each Asian/American-lineage ZIKV strain

**Fig 6:**
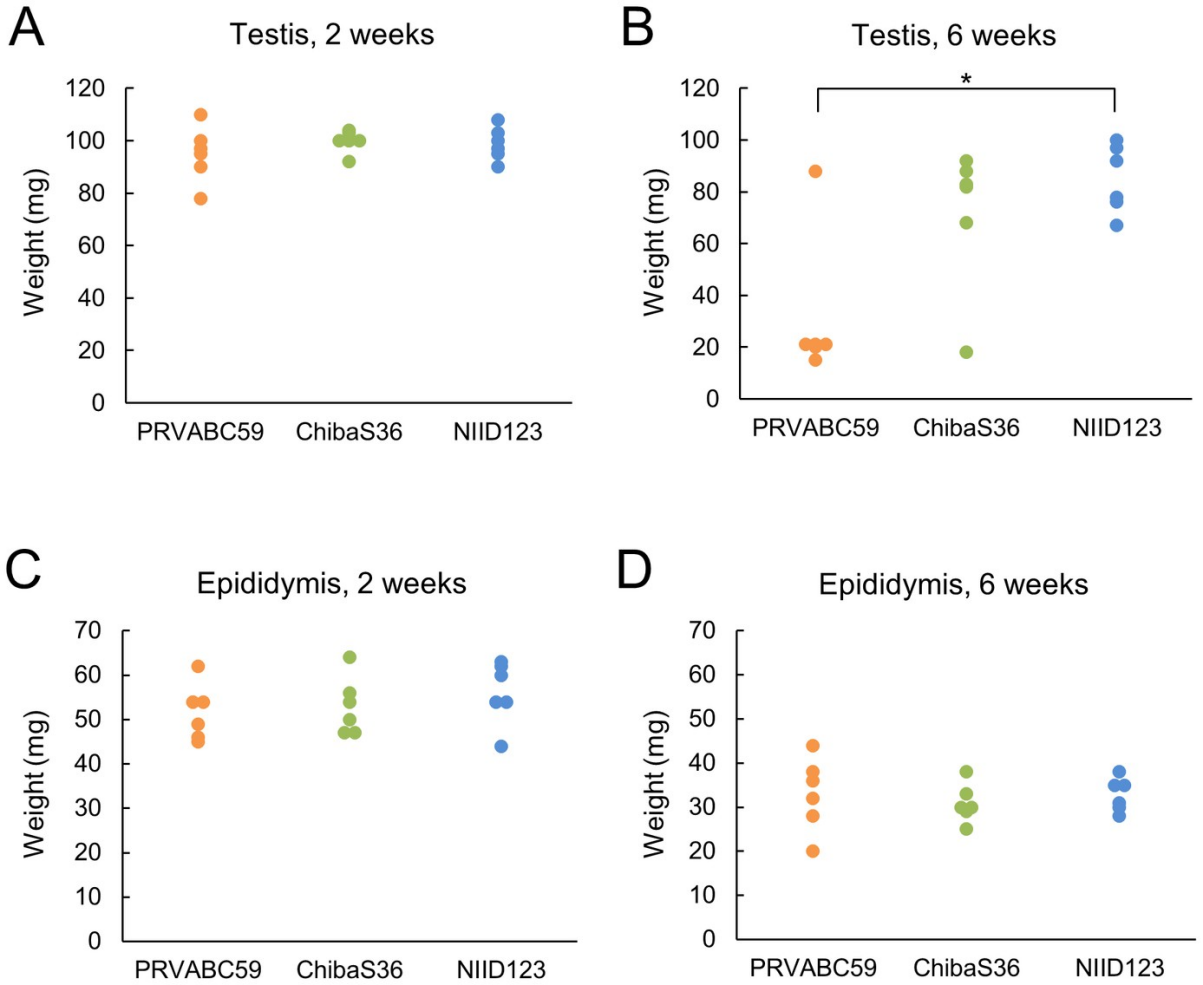
Weight of testis (A and B) and epididymis (C and D) of ZIKV-infected IFNAR1-KO mice. Mice inoculated with the Asian/American-lineage strains PRVABC59 (n = 6), ZIKV/Hu/Chiba/S36/2016 (n = 6), and ZIKV/Hu/NIID123/2016 (n = 6) were euthanized at 2 weeks (A and C) or 6 weeks (B and D) post-inoculation and the testis and epididymis were collected. Significance of differences in tissue weight was analyzed using the Mann-Whitney U test (*P < 0.05).

At 2 weeks post-inoculation, no weight loss was observed in the testis of mice infected with each ZIKV strain (Fig 6A and S2 Table). However, at 6 weeks post-inoculation, testis weight was decreased in 5/6 (83.3%) PRVABC59-infected mice and 1/6 (16.7%) ChibaS36-infected mice; the two groups of mice showed significant differences in the frequencies of testis damage (*P* = 0.04, Fisher’s exact test), but not in testis weight (Fig 6B and S2 Table). Testis weight loss was not observed in NIID123-infected mice and the frequency of testis damage was also significantly lower than that in PRVABC59-infected mice (*P* = 0.0076, Fisher’s exact test). In all three groups of ZIKV-infected mice, the epididymides were smaller at 6 weeks post-inoculation than at 2 weeks post-inoculation, but epididymis weight did not differ among the groups at the same time points (Fig 6C, 6D and S2 Table). Moreover, sperm mass was not obtained from the cauda epididymis of PRVABC59- and ChibaS36-infected mice with damaged testes.

**Fig 7:**
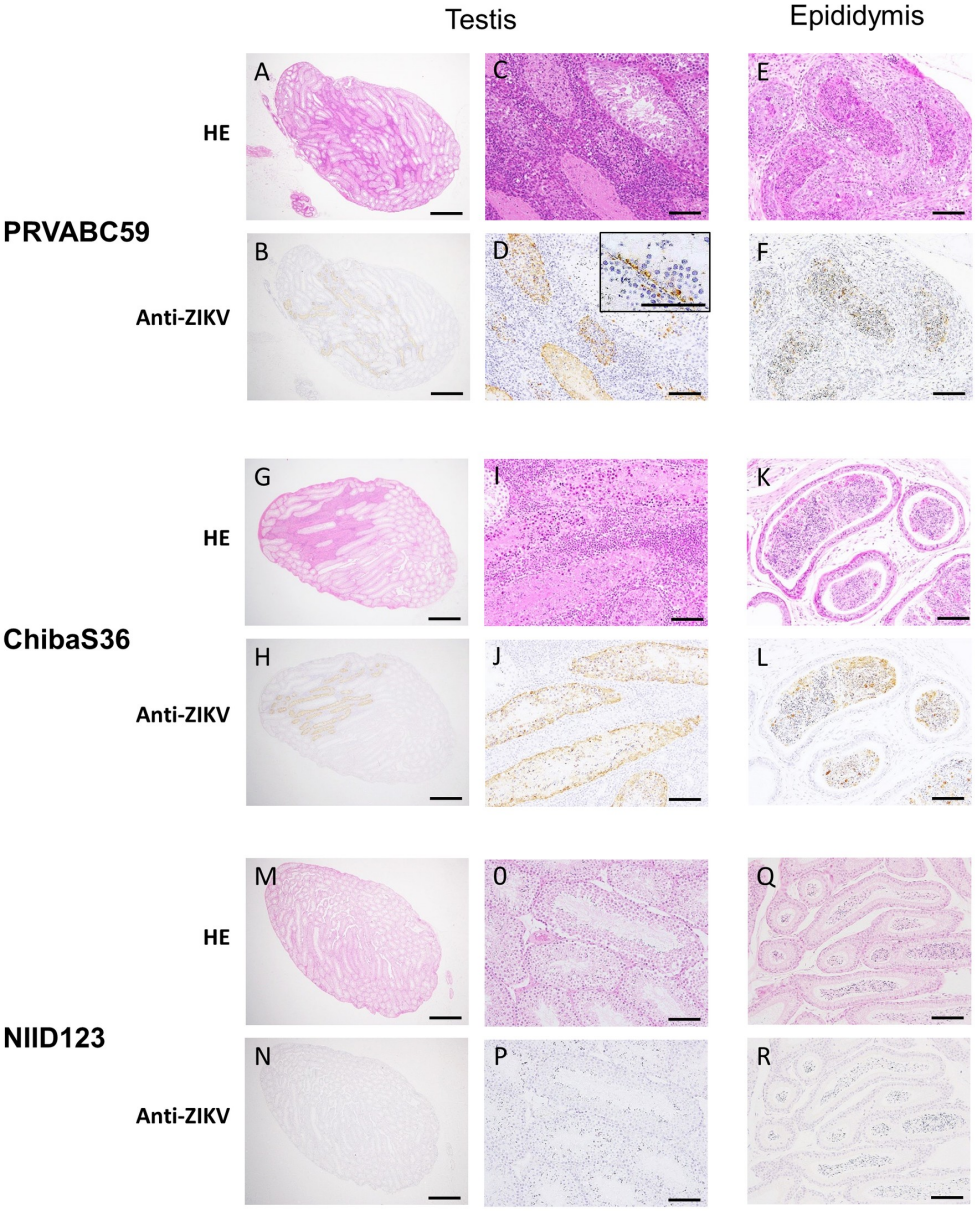
Histological analysis of testes and epididymides at 2 weeks post-inoculation. IFNAR1-KO mice were infected with PRVABC59 (A–F), ZIKV/Hu/Chiba/S36/2016 (G–L), or ZIKV/Hu/NIID123/2016 (M–R) and then euthanized at 2 weeks post-inoculation. Panel D inset: ZIKV antigen-positive cells in the damaged seminiferous tubule. Scale bars: 1,000 μm in left panels, 100 μm in center and right panels. HE: hematoxylin and eosin staining; Anti-ZIKV: immunohistochemistry of ZIKV NS1 antigen.

**Fig 8:**
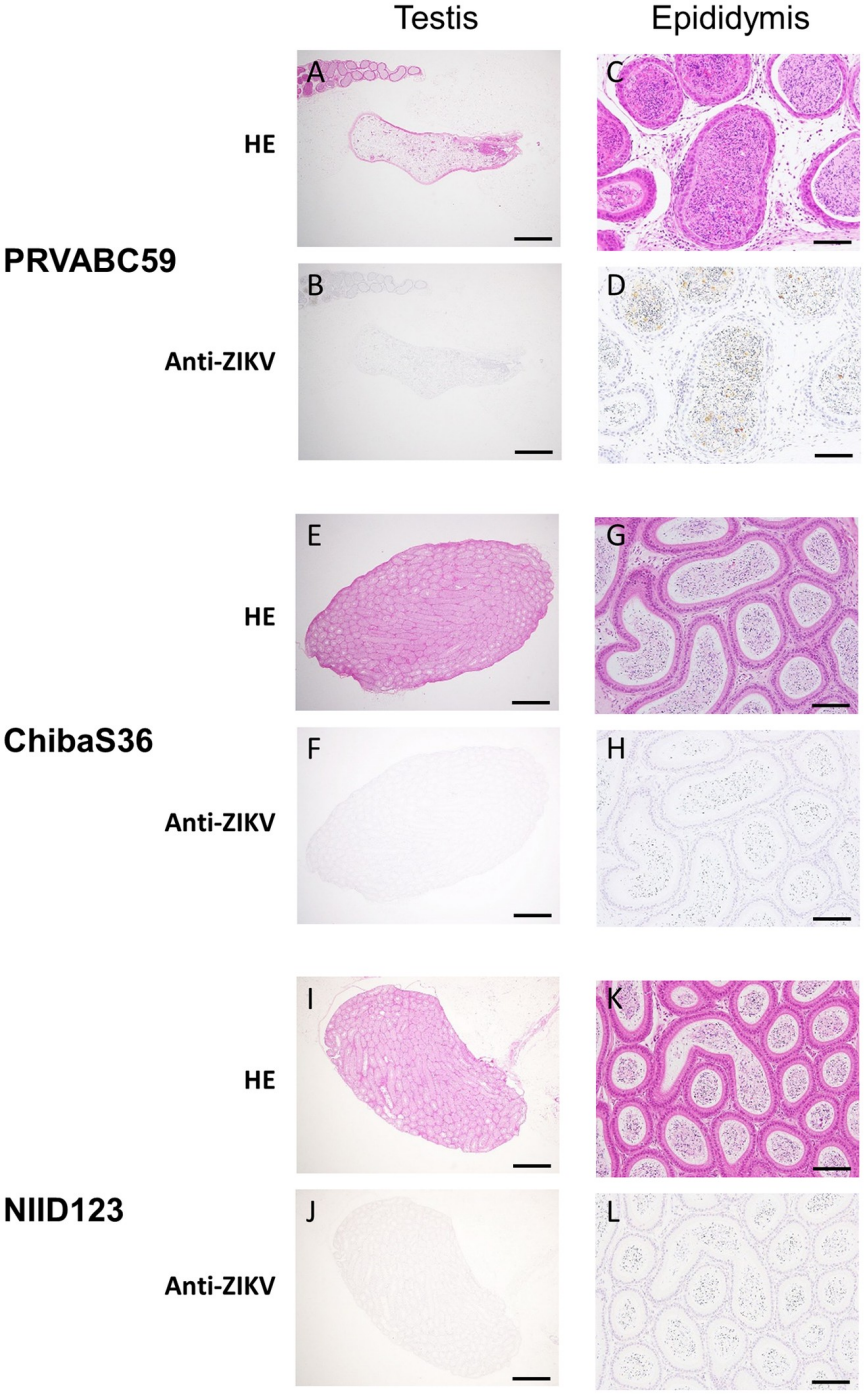
Histological analysis of testes and epididymides at 6 weeks post-inoculation. IFNAR1-KO mice were infected with PRVABC59 (A–D), ZIKV/Hu/Chiba/S36/2016 (E–H), or ZIKV/Hu/NIID123/2016 (I–L) and euthanized at 6 weeks post-inoculation. Scale bars: 1,000 μm in left panels (testes), 100 μm in right panels (epididymides). HE: hematoxylin and eosin staining; Anti-ZIKV: immunohistochemistry of ZIKV NS1 antigen.

At 2 weeks post-inoculation, germ cells were necrotic and inflammatory cells, including neutrophils, had infiltrated into the testicular interstitium in PRVABC59- and ChibaS36-infected mice (Fig 7A, 7C, 7G, and 7I). At 6 weeks post-infection, the testes of PRVABC59-infected mice were atrophic and most germ cells were not observed in the testis (Fig 8A). The ZIKV NS1 antigen was detected in the seminiferous tubules and epididymal fluid of PRVABC59- and ChibaS36-infected mice at 2 weeks post-inoculation (Fig 7B, 7D, 7F, 7H, 7J and 7L), whereas the antigen was only detected in the epididymides of PRVABC59-infected mice at 6 weeks post-inoculation (Fig 8D).

## Discussion

Previously, we reported that Asian/American-lineage ZIKV strains can be classified into three subtypes based on amino acid sequences: the Southeast Asian subtype, Pacific subtype, and American subtype [16]. In the present study we showed that ZIKV strains of these three subtypes exhibited distinct growth properties in vitro and in vivo. ZIKV NIID123, which belongs to the Southeast Asian subtype, showed lower growth capacity in vitro and in vivo and exerted a weaker injurious effect on the testis and epididymis of mice compared with the other two strains. ZIKV PRVABC59 showed the highest growth capacity both in vitro and in most of the reproductive organs examined in mice and also induced maximal damage in the testis and epididymis of the three strains. These results raise the possibility that ZIKV have acquired elevated proliferative capacity and pathogenicity during the process of the virus spreading from Southeast Asia to the Americas through the Pacific islands.

The PRVABC59 and ChibaS36 strains clearly exhibited higher growth ability than NIID123 strain in Vero cells (Fig 2B). The passage numbers of the three strains in Vero cells were ≤ 7; however, NIID123 was first passaged in C6/36 cells (Table 1). Therefore, we cannot exclude the effect of NIID123 passage history on its growth in Vero cells and mice. However, the growth rate of NIID123 in C6/36 cells was also clearly lower than PRVABC59, indicating that NIID123 has not adapted to C6/36 cells (Fig 2C).

Our results demonstrated that infection with each of the three Asian/American-subtype strains used in this study was not lethal to IFNAR1-KO mice, whereas we previously demonstrated that infection with the African-lineage MR766 strain was lethal when the mice were inoculated with a lower infectious titer (1 × 10^2^ PFU) of strain MR766 [34]. In MR766-infected mice, high levels of infectious virus and viral RNA were detected in the central nervous system at 6 days post-infection. In contrast, no infectious virus was detected in the central nervous system in mice infected with any of the Asian/American-lineage strains (Fig 3). These results support the hypothesis that the African-lineage strain causes more acute infection than the other ZIKV strains [41, 42]. However, this does not exclude the possibility that MR766 was adapted to the mouse central nervous system because of the repeated past passaging of this strain in mouse brains for virus isolation and maintenance. In this study we examined the viral loads in the brain, spinal cord, liver, kidney, and spleen in ZIKV-infected mice at only one time point (6 days post-infection); therefore, further analysis may be needed to evaluate the detailed kinetics of viral load in the organs.

At 2 weeks post-infection, the amounts of infectious particles and viral RNA in the epididymis, sperm/epididymal cells, and epididymal fluid were almost equal in PRVABC59- and ChibaS36-infected mice, but were clearly lower in NIID123-infected mice (Fig 5A and 5B). Conversely, although no infectious particles were detected at 6 weeks post-infection, higher rates and titers of the viral genome were detected in sperm/epididymal cells and epididymal fluid from PRVABC59-infected mice than from ChibaS36-infected mice (Fig 5C and 5D). These findings suggest that the American-subtype strain might be maintained for a longer period than the Pacific-subtype strain in infected mice and that the Southeast Asian-subtype strain might possess the lowest proliferative potential or might be rapidly excluded from the infected mice. At 2 weeks post-infection, the infectious virus titer and viral RNA levels in the testis were markedly high in PRVABC59-infected mice, but no infectious virus was detected in ChibaS36- and NIID123-infected mice (Fig 5A and 5B). These results raise the possibility that PRVABC59 not only induces higher levels of viremia, but also exhibits a higher ability to proliferate in the testis compared with the other two strains. At 6 weeks post-infection, viral RNA was detected in the testis in 5/6 PRVABC59-infected mice and 1/6 ChibaS36-infected mice and all the testes in which viral genomes were detected were damaged (Figs 5, 6 and 8). These data provide compelling evidence that persistent infection of testis with American- or Pacific-subtype ZIKV might represent the cause of testis damage in ZIKV-infected mice.

The observed phenotypes of the damaged testes in our experiments resembled those previously described [26, 28, 29]. The seminiferous tubule in the damaged testes showed atrophy and scarring due to germ-cell loss and inflammatory orchitis (Fig 8). In addition, we observed that the damaged testes of PRVABC59-infected mice had not recovered at 6 months post-inoculation (S2 Table), which suggests that in humans, ZIKV infection might affect reproductive ability over an extended period. However, a recent report indicated that ZIKV infection produced no clear adverse effect on morphology or hormonal production in human testis explants [43]. Moreover, no overt testis damage was observed in a primate model of ZIKV infection, suggesting that the integrity of the host immune response might be associated with the sensitivity of the testis to ZIKV infection [44].

**Fig 9:**
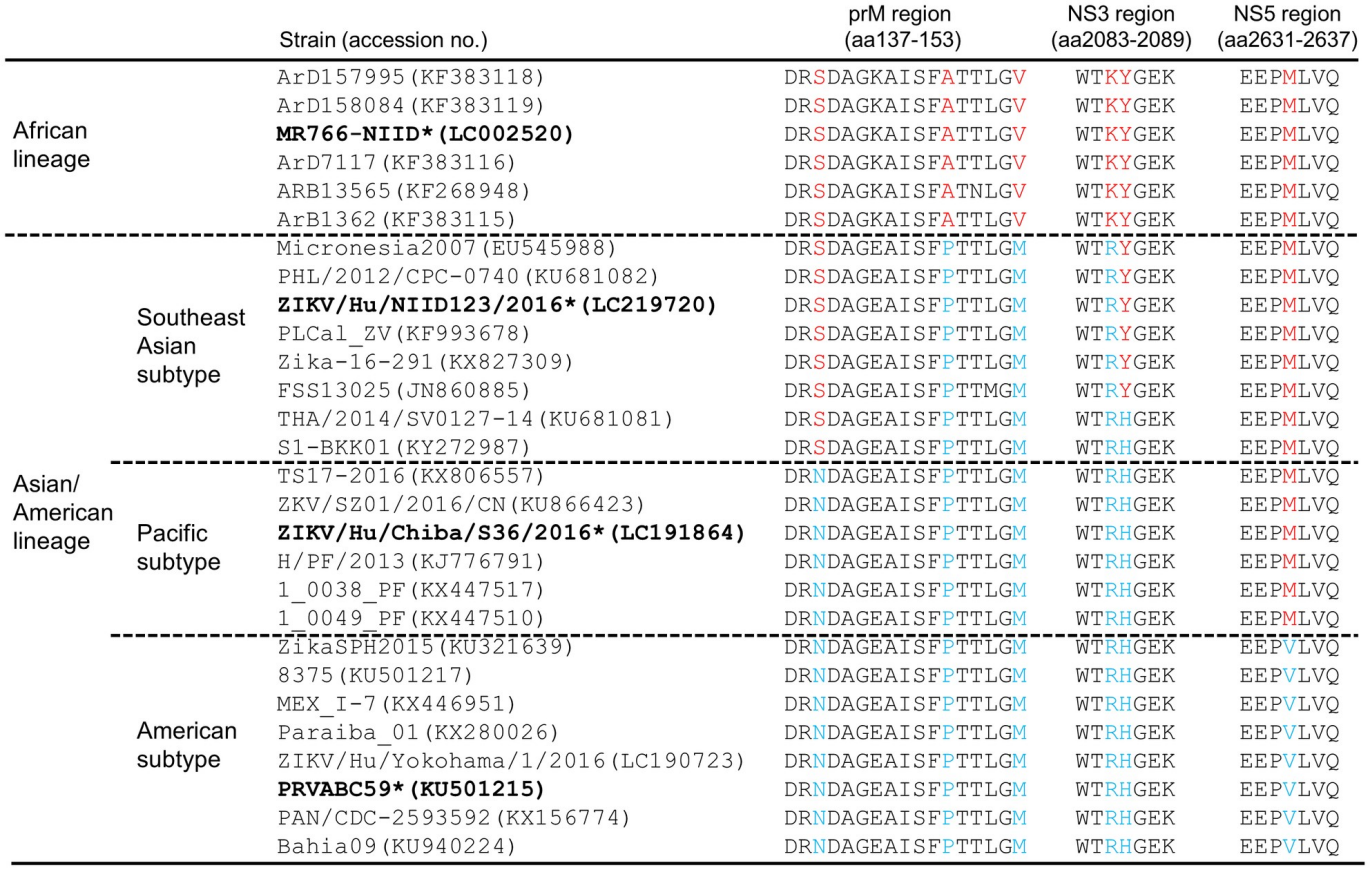
Alignment of partial amino acid sequences of ZIKV strains. Three partial sequences of the M, NS3, and NS5 regions are shown. Red/blue colors: sites of amino acid residues exhibiting variations among the strains; asterisks: strains used for characterization in this study.

Comparison of the amino acid sequences of Asian/American-subtype ZIKV strains revealed differences in several amino acid residues between the subtypes (Fig 9). The amino acid at position 139 of prM is a serine (139S) in the Southeast Asian subtype, but an asparagine (139N) in the Pacific and American subtypes. This site has been shown to be involved in the ZIKV proliferative activity in neural progenitor cells that causes microcephaly in mice; however, a recent report indicated that a confirmed congenital ZVD case with microcephaly in Thailand was caused by infection with a Southeast Asian-subtype virus harboring 139S [18, 45]. In contrast, our current results show that the Pacific and American subtypes of the virus produced clearly different pathogenic effects on the testis. However, there are distinct differences between the biological system of the fetal brain and male reproductive organs; thus, pathogenesis caused by ZIKV infection in the fetal brain does not necessarily equate with that in male reproductive organs. Therefore, factors such as variations in NS3, NS5, and other regions, which differ among the three strains, might be associated with ZIKV proliferation and the pathogenic effect on the testis and other male reproductive organs (Fig 9 and Table 2) [17, 46]. Point-by-point analysis performed using a ZIKV reverse-genetics system is required in order to identify the site(s) responsible for efficient proliferation and persistent infection of ZIKV in male reproductive organs.

**Table 2:** Amino acid residues that differ between the amino acid sequences of PRVABC59, ZIKV/Hu/Chiba/S36/2016, and ZIKV/Hu/NIID123/2016^a^.

Amino acid position	Gene	Amino acid in ZIKV strain		
		PRVABC59	ZIKV/Hu/Chiba/S36/2016	ZIKV/Hu /NIID123/2016
80	C	T	I	I
109	prM	S	N	S
139	prM	N	N	S
157	prM	Y	Y	H
193	prM	T	M	T
268	prM	L	L	F
374	E	K	K	R
550	E	S	S	N
620	E	V/L	V	V
892	NS1	W/G	W	W
893	NS1	R	R	S
900	NS1	V	V	A
975	NS1	P	S	P
1152	NS2A	M	M	I
1858	NS3	S/F	S	S
2086	NS3	H	H	Y
2197	NS4A	I	I	M
2292	NS4B	G	G	R
2611	NS5	V	A	A
2634	NS5	V	M	M
2927	NS5	N	S	N
3095	NS5	Q	H	Q
3144	NS5	N	S	N
3217	NS5	K	K	R
3391	NS5	M	M	I

^a^ The nucleotide sequences of the virus stocks used in this study were determined.

In this study, we used only one strain for each ZIKV subtype. Several variations might exist among the strains in each subtype and the strains used in the study might not necessarily reflect the properties of each subtype. Recent reports have indicated that Southeast Asian-subtype strains do not always show lower growth potential in cultured cells or lower virulence in mice compared with American-subtype strains, although the Southeast Asian-subtype strains that were used in these studies were repeatedly passaged in mouse brains [47, 48]. In contrast, Smith et al. showed that low-passage Southeast Asian-subtype strain PHL/2012/CPC-0740 exhibits high pathogenicity in IFNAR1-KO mice [49]. Thus, further evaluation must be conducted using an increased number of ZIKV strains for each subtype to elucidate the differences in characteristics among the ZIKV subtypes.

## Supporting information

S1 Table List of ZIKV strains used for phylogenetic analysis and comparison of amino acid sequences. (XLSX)

S2 Table Weight of testes and epididymides in mice inoculated with ZIKV. (PPTX)
