# Supplementary material for: Increased growth ability and pathogenicity of American- and Pacific-subtype Zika virus (ZIKV) strains compared with a Southeast Asian-subtype ZIKV strain
Source: PLoS Negl Trop Dis. 2019 Jun 6;13(6):e0007387. doi: 10.1371/journal.pntd.0007387 (PMC6553702; doi:10.1371/journal.pntd.0007387)
Supplement: S2 Table — (PPTX) [file pntd.0007387.s002.pptx]

## Slide 1
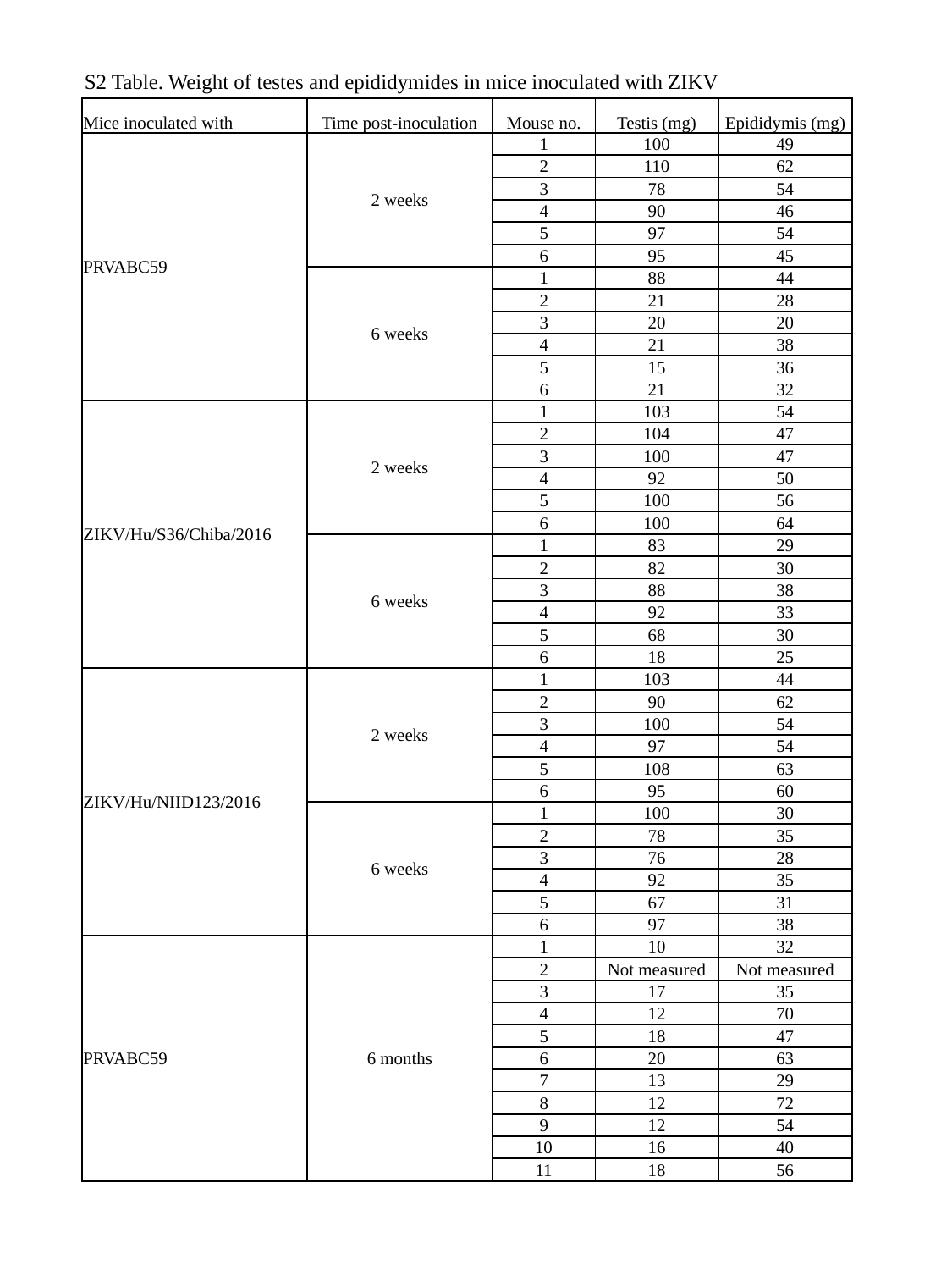

S2 Table. Weight of testes and epididymides in mice inoculated with ZIKV
| Mice inoculated with | Time post-inoculation | Mouse no. | Testis (mg) | Epididymis (mg) |
| --- | --- | --- | --- | --- |
| PRVABC59 | 2 weeks | 1 | 100 | 49 |
| | | 2 | 110 | 62 |
| | | 3 | 78 | 54 |
| | | 4 | 90 | 46 |
| | | 5 | 97 | 54 |
| | | 6 | 95 | 45 |
| | 6 weeks | 1 | 88 | 44 |
| | | 2 | 21 | 28 |
| | | 3 | 20 | 20 |
| | | 4 | 21 | 38 |
| | | 5 | 15 | 36 |
| | | 6 | 21 | 32 |
| ZIKV/Hu/S36/Chiba/2016 | 2 weeks | 1 | 103 | 54 |
| | | 2 | 104 | 47 |
| | | 3 | 100 | 47 |
| | | 4 | 92 | 50 |
| | | 5 | 100 | 56 |
| | | 6 | 100 | 64 |
| | 6 weeks | 1 | 83 | 29 |
| | | 2 | 82 | 30 |
| | | 3 | 88 | 38 |
| | | 4 | 92 | 33 |
| | | 5 | 68 | 30 |
| | | 6 | 18 | 25 |
| ZIKV/Hu/NIID123/2016 | 2 weeks | 1 | 103 | 44 |
| | | 2 | 90 | 62 |
| | | 3 | 100 | 54 |
| | | 4 | 97 | 54 |
| | | 5 | 108 | 63 |
| | | 6 | 95 | 60 |
| | 6 weeks | 1 | 100 | 30 |
| | | 2 | 78 | 35 |
| | | 3 | 76 | 28 |
| | | 4 | 92 | 35 |
| | | 5 | 67 | 31 |
| | | 6 | 97 | 38 |
| PRVABC59 | 6 months | 1 | 10 | 32 |
| | | 2 | Not measured | Not measured |
| | | 3 | 17 | 35 |
| | | 4 | 12 | 70 |
| | | 5 | 18 | 47 |
| | | 6 | 20 | 63 |
| | | 7 | 13 | 29 |
| | | 8 | 12 | 72 |
| | | 9 | 12 | 54 |
| | | 10 | 16 | 40 |
| | | 11 | 18 | 56 |
